# Patients with hypothermic sepsis have a unique gene expression profile compared to patients with fever and sepsis

**DOI:** 10.1111/jcmm.17156

**Published:** 2022-03-01

**Authors:** Matthew B. A. Harmon, Brendon P. Scicluna, Maryse A. Wiewel, Marcus J. Schultz, Janneke Horn, Olaf L. Cremer, Tom van der Poll, W. Joost Wiersinga, Nicole P. Juffermans, the MARS consortium, Friso M de Beer, Lieuwe D Bos, Gerie J Glas, Janneke Horn, Arie J Hoogendijk, Roosmarijn T van Hooijdonk, Mischa A Huson, Tom van der Poll, Brendon Scicluna, Laura R Schouten, Marcus J Schultz, Marleen Straat, Lonneke A. van Vught, Luuk Wieske, Maryse A Wiewel, Esther Witteveen, Marc J Bonten, Olaf L Cremer, Jos F Frencken, Kirsten van de Groep, Peter M Klein Klouwenberg, Maria E Koster–Brouwer, David S Ong, Meri R Varkila, Diana M Verboom

**Affiliations:** ^1^ Department of Intensive Care Amsterdam University Medical Centers location Academic Medical Centre University of Amsterdam Amsterdam The Netherlands; ^2^ Laboratory of Experimental Intensive Care and Anesthesiology Amsterdam University Medical Centers location Academic Medical Centre University of Amsterdam Amsterdam The Netherlands; ^3^ Center for Experimental & Molecular Medicine Amsterdam University Medical Centers location Academic Medical Center University of Amsterdam Amsterdam The Netherlands; ^4^ Mahidol Oxford Research Unit Mahidol University Bangkok Thailand; ^5^ Nuffield Department of Medicine University of Oxford Oxford UK; ^6^ Department of Intensive Care Medicine University Medical Center Utrecht Utrecht The Netherlands

**Keywords:** biomarker, fever, hypothermia, microarray, sepsis, tryptophan

## Abstract

The pathophysiology of hypothermia during sepsis is unclear. Using genomic profiling of blood leukocytes, we aimed to determine if hypothermia is associated with a different gene expression profile compared to fever during sepsis. Patients with sepsis and either hypothermia or fever within 24 hours after ICU admission were included in the study (*n* = 168). Hypothermia was defined as body temperature below 36 °C. Fever was defined as body temperature equal to or above 38.3°C. We compared blood gene expression (whole‐genome transcriptome in leukocytes) in hypothermic septic compared to febrile septic patients in an unmatched analysis and matched for APACHE IV score and the presence of shock. In total, 67 septic patients were hypothermic and 101 patients were febrile. Hypothermia was associated with a distinct gene expression profile in both unmatched and matched analyses. There were significant differences related to the up‐ and downregulation of canonical signalling pathways. In the matched analysis, the top upregulated gene was cold‐inducible mRNA binding protein (CIRBP) which plays a role in cold‐induced suppression of cell proliferation. In addition, we found three signalling pathways significantly upregulated in hypothermic patients compared to febrile patients; tryptophan degradation X, phenylalanine degradation IV and putrescine degradation III. In conclusion, there are distinct signalling pathways and genes associated with hypothermia, including tryptophan degradation and CIRBP expression, providing a possible link to the modulation of body temperature and early immunosuppression. Future studies may focus on the canonical signalling pathways presented in this paper to further investigate spontaneous hypothermia in sepsis.

## 1 INTRODUCTION

Body temperature changes are common in sepsis.^1^ Patients who present with spontaneous hypothermia suffer from substantially increased morbidity and mortality compared to their normothermic or febrile counterparts.^2^, ^3^ It is unclear whether hypothermia simply represents a symptom of severe inflammation or that hypothermia itself drives mortality through a yet unknown mechanism.^4^, ^5^ Animal studies even indicate that hypothermia may be an adaptive response to severe inflammation in order to limit metabolism and prevent hypoxia.^6^

To determine the aetiology of the hypothermic response during sepsis, studies have mainly focused on the ability to generate an adequate host immune response, often with levels of pro‐inflammatory cytokines as a read‐out. However, studies have not confirmed a defective immune host response, as septic patients with hypothermia had similar,^7^, ^8^ or even increased proinflammatory cytokine levels^9^ in comparison with normothermic or febrile patients. Patients with hypothermic sepsis do develop persistent lymphopenia, a marker of immunosuppression.^10^ Alternatively, the cardiovascular system may play a role. We previously showed that markers of endothelial injury are increased in hypothermic sepsis compared to nonhypothermic controls.^7^

Taken together, the pathophysiology of hypothermic response in sepsis remains ill defined. Whole blood transcriptome analysis has provided valuable insights in the complex pathophysiology of the sepsis syndrome.^11^ In this study, we aimed to determine if hypothermia in sepsis patients is associated with a different blood leukocyte gene expression profile compared to fever. We hypothesized that the blood transcriptomes of hypothermic sepsis patients differed from those obtained in febrile sepsis patients, which in turn reflect on variations in the host response.

## 2 MATERIALS AND METHODS

### 2.1 Study design, setting and patient identification

This study was performed within the Molecular Diagnosis and Risk Stratification of Sepsis (MARS) project, a prospective observational cohort study in mixed ICUs of two tertiary teaching hospitals (Academic Medical Center in Amsterdam and University Medical Center in Utrecht) in the Netherlands (ClinicalTrials.gov identifier NCT01905033).^12^, ^13^ Between January 2011 and July 2012, patients older than 18 years of age with an expected length of stay longer than 24 h were included via an opt‐out consent method approved by ethical committees of both hospitals (IRB no. 10‐056). During this study, demographic, clinical, microbiology and interventional data were collected daily by trained research physicians. The plausibility of an infection was assessed using a four‐point scale (none, possible, probable or definite) using Centers for Disease Control and Prevention and International Sepsis Forum consensus definitions^14^, ^15^ as described previously.^12^

We included patients diagnosed with sepsis and having blood microarray data obtained within the first 24 h of ICU admission. Sepsis was defined on ICU admission as having definite or probable infection,^12^ combined with at least one parameter of inflammatory dysfunction, hemodynamic dysfunction, organ dysfunction or deranged tissue perfusion.^16^ To limit the occurrence of iatrogenic hypothermia, patients admitted from the operating room (OR), ICU readmissions, patients undergoing active cooling, patients transferred from another ICU and patients with immunosuppression were excluded from this study.^7^ Shock was defined as hypotension requiring treatment with vasopressors at a dose of 0.1 mcg/kg/min during at least 50% of the day. Clinical severity was assessed by Acute Physiology and Chronic Health Evaluation (APACHE) IV and Sequential Organ Failure Assessment (SOFA) scores. Temperature was removed from the APACHE IV scores which are presented in the tables and subsequent matched analyses.

Body temperatures were prospectively validated every hour by the treating ICU nurse and subsequently the researcher. To control for any body temperatures that may have been inadvertently entered in the database (i.e. a rectal sensor that has been displaced and is exposed to ambient temperature), patients with unreliably low measurements of temperature (below 33°C) were excluded. Also, patients with only one registered temperature measurement during the first 24 h were not included. Temperature was measured using a rectal, nasal, inguinal or tympanic temperature probes. Core temperatures were used in preference to inguinal or tympanic measurements.

Hypothermia was defined as a minimum body temperature <36.0°C and a maximum body temperature <38.3°C in the first 24 h of ICU admission. Conversely, fever was defined as a maximum body temperature ≥38.3°C and a minimum body temperature ≥36.0°C as defined in previous studies.^10^, ^17^ Normothermia was defined a minimum and maximum body temperature between ≥36.0°C and <38.3°C. Patients were defined as “both” if they had both hypothermia and fever in the first 24 h of ICU admission.

### 2.2 Blood gene expression microarrays and bioinformatics

Whole blood was collected in PAXgene tubes (Becton‐Dickinson) within 24 h after ICU admission and total RNA was isolated using the PAXgene blood mRNA kit (Qiagen, Venlo, the Netherlands) in combination with QIAcube automated system (Qiagen), as previously described.^13^, ^18^, ^19^ The RNA isolation excluded material from erythrocytes. Microarray data (Affymetrix Human Genome U219 96‐array plates) are accessible to the public via the National Center for Biotechnology Information (NCBI) Gene Expression Omnibus (GEO) accession GSE65682. Briefly, raw scans were pre‐processed by means of the robust multi‐average (RMA) method, normalized (quantile), summarized by median polish and log2 transformed using the affy method.^20^ Nonexperimental chip effects were assessed and corrected by means of the combat method in the surrogate variable analysis R package.^21^ Comparisons between groups were done using multi‐variate linear models, including age and gender as covariates, implemented in the limma method.^22^ Benjamini–Hochberg (BH)‐adjusted *p*‐values <0.05 defined genome‐wide significance. To assess the association with canonical signalling pathways, we used Ingenuity Pathway Analysis software (Qiagen Bioinformatics). Fisher exact test BH‐adjusted *p*‐values <0.05 demarcated significance. Human species and Ingenuity gene knowledgebase were specified. All other parameters were default.

### 2.3 Statistical analysis

All data were analysed using R studio (version 3.2.2, R Core Team 2013, Vienna, Austria). In this study, we performed two analyses. Firstly, we compared genomic profiles between septic hypothermic and febrile patients without correcting for disease severity. Subsequently, in order to determine whether hypothermia was associated with a specific genomic profile irrespective of severity of disease, hypothermic patients were 1:1 matched to fever patients using their APACHE IV scores and presence of shock. Data are presented as numbers (percentages), parametric data as mean ± SD and nonparametric data as median and 25th–75th percentages; Q1–Q3. Data distribution was assessed by the Kolmogorov–Smirnov test. Mann–Whitney U or a Kruskal–Wallis test was used to analyse continuous nonparametric data, whereas continuous parametric data were analysed using Student's *t*‐test or analysis of variance (two‐sided analysis of variance). All categorical data were analysed using a chi‐square or Fisher exact test. A *p*‐value less than 0.05 was considered to be of statistical significance for clinical data. Matching was done using “optimal matching” with R‐package “MatchIt” (calliper 0.35 standard deviations of the logit), which locates matched samples with the smallest average absolute distance across all matched pairs.

## 3 RESULTS

### 3.1 Patients

**FIGURE 1:**
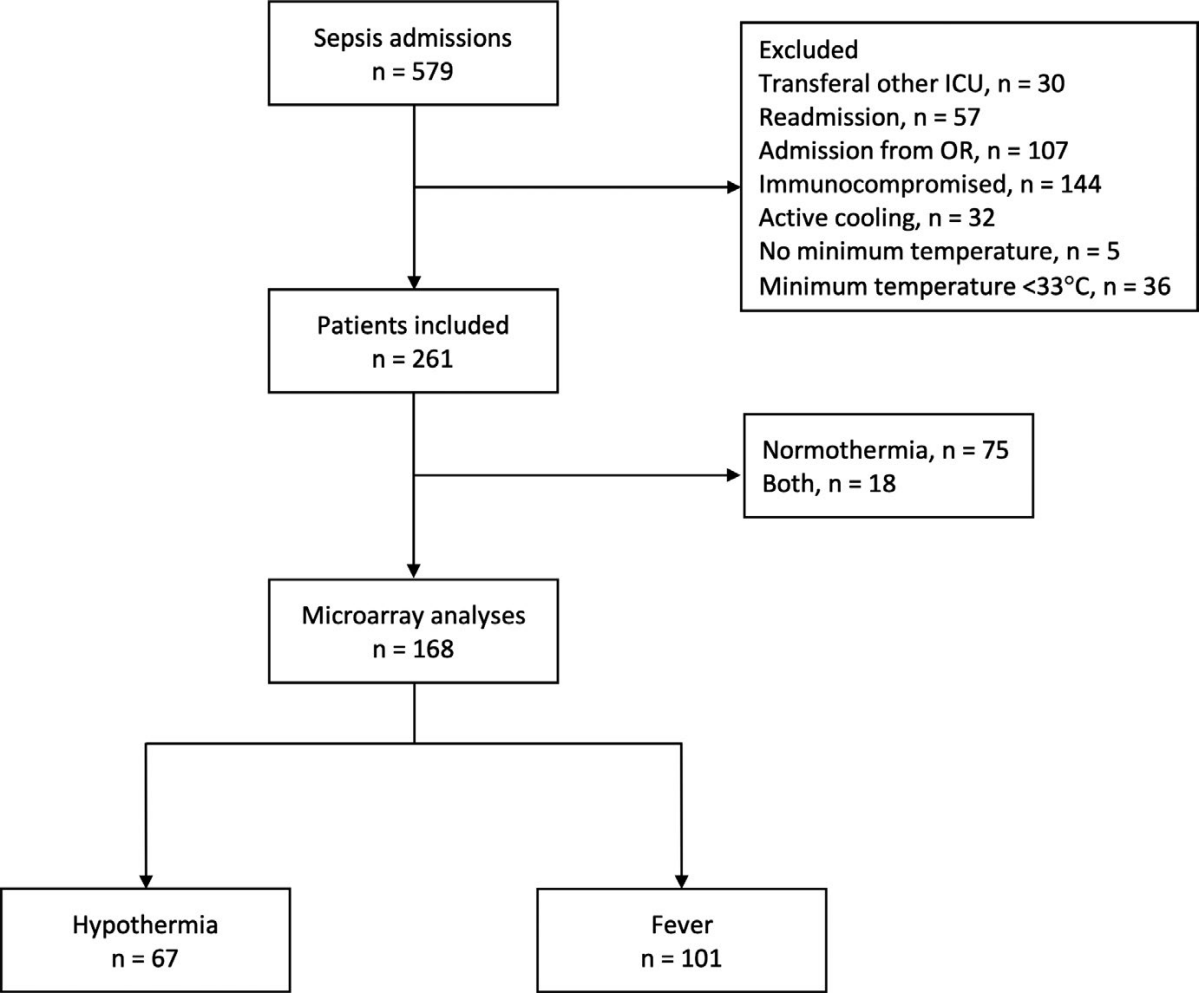
Flowchart showing the selection of study patients. (Some patients were excluded for multiple reasons and were counted multiple times for reason of exclusion).

The selection of study patients can be seen in Figure 1. Out of a total of 579 sepsis admissions, 168 patients were included in the microarray analysis. Table 1 shows the baseline characteristics of these patients arranged by temperature group (baseline characteristics of patients that were normothermic (*n* = 75) or both hypothermic and febrile (*n* = 18) are shown in Table S1 for the purpose of interpretation, but these were not included in the microarray analysis).

**TABLE 1:** Baseline characteristics and outcome of sepsis patients with hypothermia vs. fever

	Hypothermia *N* = 67	Fever *N* = 101	*p*‐value
Demographics			
Age, years, mean [SD]	68.1 [10.9]	60.0 [16.6]	<0.0001
Gender, male (%)	37 (55)	66 (65)	0.215
BMI, kg/m^2^, mean [SD]	25.5 [5.5]	27.0 [7.3]	0.155
Comorbidities			
Charlson score, median [IQR]	5 [3–6]	4 [2–6]	0.003
Chronic cardiovascular insufficiency (%)	4 (6)	2 (2)	0.208
Chronic renal insufficiency (%)	11 (16)	6 (6)	0.041
Congestive heart failure (%)	3 (4)	4 (4)	1
COPD (%)	9 (13)	17 (17)	0.653
Diabetes mellitus (%)	19 (28)	16 (16)	0.051
Site of infection			
Pulmonary (%)	28 (42)	50 (50)	0.450
Abdominal (%)	13 (19)	21 (21)	‐
Urinary tract (%)	10 (15)	9 (9)	‐
Other (%)	3 (4)	8 (8)	‐
Co‐infection (%)	13 (19)	13 (13)	‐
Severity of disease first 24 h			
Min temp first 24 h, mean [SD]	35.0 [0.9]	37.2 [0.7]	<0.0001
Max temp first 24 h, mean [SD]	37.1 [0.9]	39.3 [0.9]	<0.0001
APACHE IV score, median [IQR]^a^	82 [71.5–104.5]	68 [55–84]	<0.0001
SOFA score, median [IQR]^b^	9 [6–11]	7 [4–8]	<0.0001
Acute kidney injury (%)	38 (57)	31 (31)	0.001
Renal replacement therapy (%)	14 (21)	8 (8)	0.021
Acute lung injury (%)	21 (31)	30 (30)	0.864
Shock (%)	32 (48)	25 (25)	0.001
Clinical laboratory parameters first 24 h			
WBC count max. (x10^9^/L), median [IQR]	17.4 [10.6–27.8]	14.0 [9.9–17.9]	0.06
Platelets min. (x10^9^/L), median [IQR]	186 [114–254]	208 [131–305]	0.112
Lactate max. (mmol/L), median [IQR]	3.2 [2.1–9.1]	2.5 [1.6–3.7]	0.011
Prothrombin time max. (s), median [IQR]	16.3 [14.0–22–2]	14.1 [12.1–16.8]	<0.0001
Creatinine max. (μmol/L), median [IQR]	114 [76–200]	99 [71.5–161.5]	0.114
Outcome			
ICU‐mortality (%)	21 (31)	9 (9)	<0.0001
30‐day mortality (%)	29 (43)	16 (16)	<0.0001
90‐day mortality (%)	35 (52)	22 (22)	0.001

^a^ Temperature not included in score.

^b^ Central nervous system not included in score due to large number of sedated patients.

Abbreviations: APACHE, acute physiology and chronic health evaluation; COPD, chronic obstructive pulmonary disease; IQR, interquartile range; SD, standard deviation; SOFA, sequential organ failure assessment; WBC, white blood cell.

Of the 168 included patients, 67 patients were hypothermic and 101 patients were febrile. Minimum temperature in the hypothermic group was lower compared to the febrile group (35.0°C ± 0.9 *vs*. 37.2°C ± 0.7) as was the maximum temperature (37.1°C ± 0.9 *vs*. 39.3°C ± 0.9). Hypothermic patients were also older compared to febrile patients, but BMI and gender distribution were similar between groups. There was also a similar distribution of site of infection between hypothermic and febrile patients.

Patients in the hypothermic group compared to febrile patients had higher APACHE IV scores and SOFA scores, increased incidence of shock and higher rates of mortality at 30 days post‐ICU admission (29 (43%) *vs*. 22 (22%)).

### 3.2 Alterations in microarray gene expression in hypothermia compared to fever

**FIGURE 2:**
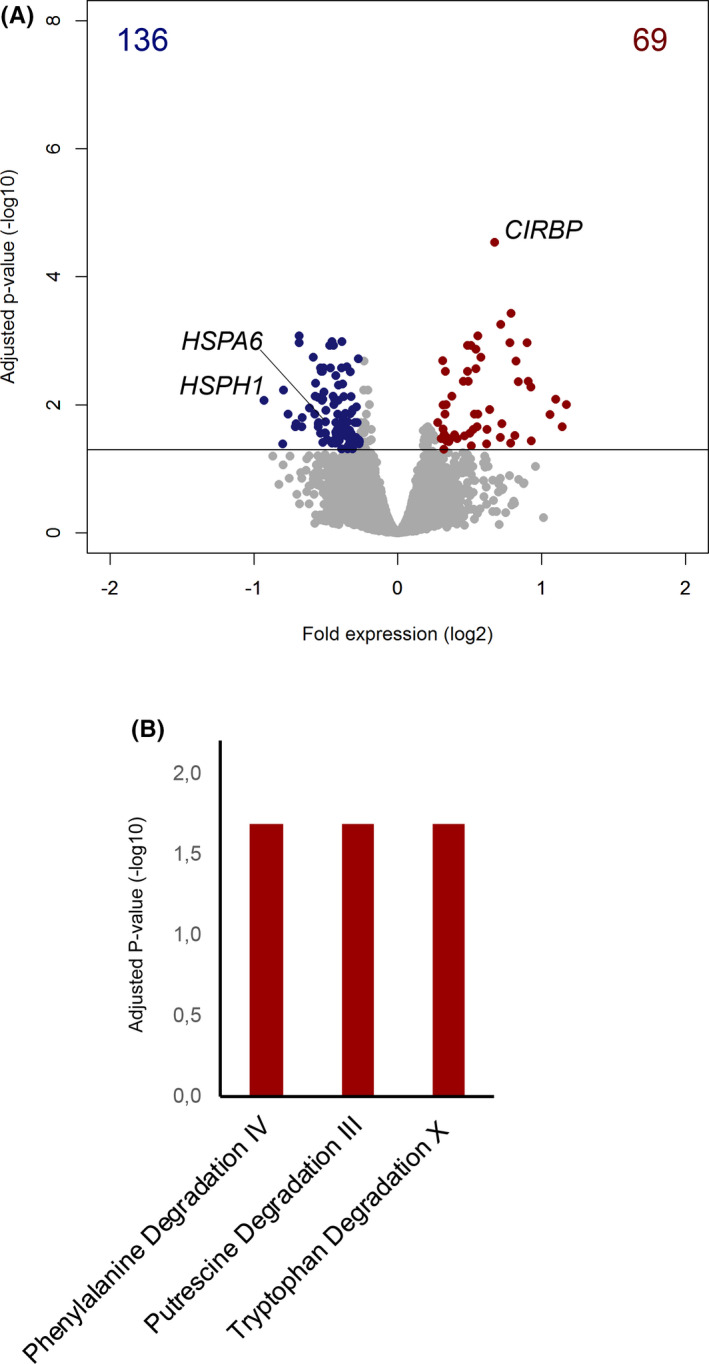
Gene expression profiles from whole blood leukocyte microarray analysis in hypothermic septic patients versus febrile septic patients. (A). Volcano plot (integrating adjusted *p*‐values and fold expression indices) of gene expression differences in hypothermic septic patients compared to febrile patients in a cohort matched for APACHE IV scores and shock. Red dots, high expression genes; blue dots, low expression genes. HSPA6, HSPH1 and CIRBP are highlighted in this figure as their expression is temperature dependent. A comprehensive list of differentially expressed protein‐coding genes is tabulated in Table S2. (B) Bar plot showing high expression genes significantly associated with Ingenuity's canonical signalling pathways in hypothermia compared to fever.

A total of 1930 transcripts were significantly altered in hypothermic patients compared to febrile patients, of which 1425 were reduced and 505 transcripts were elevated (Figures S1 and S2A). Figure S2B shows the significant canonical pathways associated with these genes. Subsequently, we matched patients for APACHE IV scores and presence of shock. In total, 55 patients in each group remained for further analysis (characteristics of whom are shown in Table S2). After matching, there was no difference between groups in terms of APACHE IV scores and SOFA scores as well as incidence of shock. The 30‐day mortality remained significantly increased in hypothermic patients (21 (38%) *vs*. 10 (18%), *p* = 0.041). Despite matching for APACHE IV scores, which includes age, patients in the hypothermic group were significantly older (67.3 years±11.6 vs. 61.8 years±16.3, *p* = 0.044). In total, 205 transcripts were significantly altered in hypothermic patients compared to febrile patients, of which 136 were reduced and 69 were elevated (Figure 2A). The top‐most significant gene was *CIRBP*, encoding cold‐induced RNA binding protein, which plays a role in cold‐induced suppression of cell proliferation. Among the genes with decreased expression in hypothermic patients, we found members of the heat shock protein 70 complex (HSP70), namely *HSPH1* and *HSPA6*, encoding chaperone proteins that play essential roles in stress‐induced misfolded protein responses (Figure 2A). These genes have been highlighted as their expression is temperature dependent and provide a genetic validation of temperature differences between groups. A comprehensive list of differentially expressed protein‐coding genes is tabulated in Table S3. Pathway analysis of significantly altered, high expression genes resulted in significant associations with tryptophan degradation X, phenylalanine degradation IV and putrescine degradation III canonical signalling pathways (Figure 2B). These signalling pathways relate to immunometabolic reactions that function in the degradation of amino acids and (poly)amines.

## 4 DISCUSSION

In this study of canonical pathways in blood leukocytes in septic patients, we found that hypothermic septic patients have a unique gene expression profile compared to sepsis patient presenting with fever. After correcting for disease severity, hypothermic septic patients showed a surprisingly similar gene expression profile compared patients with febrile sepsis. However, there were distinct upregulated signalling pathways related to degradation of amino acids and (poly)amines were strongly associated with hypothermia, both in uncorrected analyses and analyses corrected for disease severity.

Patients with hypothermic sepsis show significant alterations in genomic pathways compared to febrile patients. These included downregulated pathways related to protein catabolism and translation, cell growth proliferation and mobility, cardiovascular signalling, pattern recognition receptor and cytokine signalling and lymphocyte pathways. In addition, pathways relating to amino acid and (poly)amine degradation were significantly upregulated. In a different sepsis cohort, similar defects in metabolic and immunologic signalling pathways have been associated with underlying immunoparalysis encountered in the acute phase of an infection.^23^ In line with this finding, a recent retrospective analysis of hypothermic septic patients found increased incidence of lymphopenia associated with hypothermia compared to nonhypothermic septic patients.^10^ However, other studies focusing on the host response have revealed remarkably few differences between hypothermic and nonhypothermic or febrile patients regarding typical pro‐ and anti‐inflammatory cytokine responses and thereby signal that hypothermic patients are initially able to mount an adequate host response.^7^, ^8^

It is important to note that on the basis of blood transcriptomic data alone, the presence of generalized sepsis immunosuppression is not definite. Patterns of leukocyte gene expression may represent a state of cellular reprogramming attuned to an inflammatory resolution phase concomitant with an increased anti‐microbial response.^24^, ^25^ Therefore, the hyporesponsive characteristics of immune cells observed both in vitro and in vivo, which have been often perceived as a sign of deleterious immunosuppression, may constitute normal functional reprogramming of immune cells that is not harmful to the host by promoting pathogen elimination and tissue recovery.^26^

Subsequently, to address confounding due to increased disease severity in hypothermic patients, we adjusted our model for illness severity by matching of patients on APACHE IV score and presence of shock. In this analysis, many of the identified genetic pathways found in the unmatched cohort were no longer present in the matched cohort, suggesting that these pathways may be related to disease severity.

Cold‐inducible mRNA binding protein expression, which encodes cold‐induced RNA binding protein, was significantly elevated in patients discordant for hypothermia sepsis, importantly, after matching patients for disease severity. CIRBP expression is altered in several different species in response to lower temperatures, suggesting that CIRBP is a conserved response to cold stress.^27^ CIRPB expression is upregulated during hypoxia, hypothermia and oxidative stress.^28^ It plays a role in cold‐induced cell suppression. In addition, it can trigger an inflammatory response in sepsis^29^ and may be a critical mediator in organ failure during sepsis.^28^ HSPH1 and HSPA6, members of the HSP70 complex, were downregulated in hypothermic septic patients. HSP70 expression is temperature dependent and important for sustaining immune function during sepsis as well as cell protection.^30^ Among other things, it can upregulate the expression of proinflammatory and pyrogenic cytokines, such as tumour necrosis factor alpha and interleukin‐1 beta.^31^ HSP70 deficiency can aggravate peritonitis in mice.^32^

In the matched analysis, three canonical pathways associated with amino acid and polyamine degradation were significantly upregulated in hypothermic septic patients compared to febrile septic patients. First, the tryptophan degradation X pathway was upregulated. Tryptophan.^33^ Metabolites regulate anti‐inflammatory effects of the immune response.^33^, ^34^ In sepsis, increased degradation from tryptophan to kynurenine is associated with decreased lymphocyte counts.^35^ In vitro and in vivo evidence shows that tryptophan metabolites are fatal for T‐cell survival.^36^ Tryptophan is also degraded to serotonin. Interestingly, serotonin deficient mice are extremely susceptible to temperature variations and show a profound hypothermic response when placed in a cold environment.^37^ Also, serotonin receptor antagonists has improved survival in experimental settings, but has not been evaluated in humans.^38^

Secondly, the putrescine degradation III pathway was upregulated. Putrescine has shown the potential to modulate the innate immune response.^39^ In a retrospective study in patients with community acquired pneumonia, putrescine levels were associated with disease severity and mortality.^40^ In addition, putrescine is essential to the survival of important pathogens such as *Streptococcus pneumoniae* ^41^, ^42^ and *Escherischia coli*.^43^

Phenylalanine degradation IV pathway was the third pathway which was significantly upregulated; phenylalanine is a precursor to L‐dopa and catecholamines. Increased serum levels of phenylalanine have been shown to be increased in patients after trauma, burns and sepsis^44^, ^45^ and predict mortality in patients with a severe infection.^46^ Increased serum levels of phenylalanine are related to insufficient tissue perfusion and impaired cellular energy production. Impaired phenylalanine metabolism can interfere with the production of catecholamine and augment shock.^45^

In general, the aetiology and pathophysiology of the hypothermic response remains unclear. Our study suggests that patients with hypothermia have distinct differences in gene expression profiles. Several pathways were altered in the unmatched analysis but not in the matched analysis, suggesting that these pathways are not specific to hypothermia. However, by matching for disease severity, important pathways relating hypothermia and sepsis may be overlooked as hypothermia may represent a symptom of disease severity.^47^ How the genes and pathways found in this study relate to the hypothermic response and increased mortality remains to be seen.

This study has several strengths. In this prospective cohort, we looked at the extremes of temperatures in sepsis (hypothermia *vs*. fever). By doing this, we reason we had the highest likelihood of finding pathways that relate to the hypothermic septic response. Of note, our study is based on a prospective observational cohort that was not designed to address the scope of our study. Future studies in larger cohorts of sepsis with or without hypothermia are certainly warranted. Secondly, instead of using conventional biomarkers, we used microarray in blood leukocytes to identify potential clues to the aetiology of the hypothermic response in sepsis. Blood leukocytes represent a clinically relevant and easily accessible body compartment that has been extensively employed in clinical studies to identify fundamental features of the immune response during sepsis.^11^ Results from this study can guide future studies on the pathophysiology of the hypothermic response.

There are also several limitations to this study. First of all, pathway analysis based on transcriptional profiles does not clarify whether the upregulation or downregulation of pathways indicates a lack, or overabundance of a specific protein substrate. Future studies on the metabolite and protein products of the herein inferred canonical signalling pathways are warranted. Second, we did not standardize the timing and method of temperature measurements. Information on the site and frequency of temperature measurements would have ideally been included in this study. For example, it is however, core temperature measurements are common practice in the ICU setting, we controlled for potentially incorrect temperature measurements and the matched analysis likely corrects for any imbalances in measurements between groups. Furthermore, in this analysis we chose only to compare hypothermic patients to those with fever. As a result, there are large temperature differences between these two groups potentially minimizing the effect of any measurement inaccuracies. Also, blood sampling was performed in the first 24 h of ICU admission. The sampling did not necessarily coincide with the hypothermic or febrile temperature measurement. However, we do not think that sampling needs to be simultaneous with temperature measurements in order to characterize this group of patients. A single hypothermic measurement in the first 24 h of ICU admission is significantly associated with adverse outcome,^1^, ^7^ and changes associated with hypothermia likely persist beyond the hypothermic measurement. Finally, in the matched analysis, age was significantly higher in the hypothermic group compared to the febrile group, despite being in the APACHE IV score. Though age (and gender) was added as a covariate in our transcriptome analysis, the former may confound results, at least in part, since prior studies have shown that age is an independent risk factor for hypothermia.^48^ Finally, due to the inherent nature of observational studies, cause–effect relationships cannot be established.

In conclusion, hypothermic patients were characterized by largely similar, but also significant changes in leukocyte transcriptomes. Genes were associated with distinct cellular biological pathways, including tryptophan metabolism and serotonin‐signalling. Cold‐inducible mRNA binding protein (CIRBP) expression was particularly elevated in sepsis patients with hypothermia. These signalling pathways provide a possible link to the modulation of body temperature and early immunosuppression. Future functional studies on the canonical signalling pathways and specific genes presented in this paper are warranted.

## CONFLICT OF INTEREST

The authors of this study declare no conflict of interest.

## AUTHOR CONTRIBUTION

**Matthew Harmon:** Conceptualization (lead); Data curation (equal); Formal analysis (equal); Methodology (equal); Writing – original draft (lead); Writing – review & editing (lead). **Brendon P. Scicluna:** Data curation (lead); Formal analysis (lead); Investigation (equal); Methodology (equal); Writing – review & editing (equal). **Maryse A. Wiewel:** Conceptualization (equal); Data curation (equal); Investigation (equal); Methodology (equal); Writing – review & editing (equal). **Marcus J. Schultz:** Conceptualization (equal); Methodology (equal); Writing – review & editing (equal). **Janneke Horn:** Methodology (equal); Writing – review & editing (equal). **Olaf Cremer:** Conceptualization (equal); Investigation (equal); Methodology (equal); Writing – review & editing (equal). **Tom van der Poll:** Conceptualization (lead); Funding acquisition (lead); Investigation (equal); Methodology (lead); Resources (lead); Supervision (equal); Writing – review & editing (equal). **Joost Wiersinga:** Conceptualization (equal); Investigation (equal); Methodology (equal); Writing – review & editing (equal). **Nicole Juffermans:** Conceptualization (lead); Investigation (equal); Methodology (lead); Writing – original draft (lead); Writing – review & editing (lead).

## Supporting information

Supplementary Material

## Appendix

MARS consortium: Friso M. de Beer, Lieuwe D. Bos, Gerie J. Glas, Janneke Horn, Arie J. Hoogendijk, Roosmarijn T. van Hooijdonk, Mischa A. Huson, Tom van der Poll, Brendon P. Scicluna, Laura R. Schouten, Marcus J. Schultz, Marleen Straat, Lonneke A. van Vught, Luuk Wieske, Maryse A. Wiewel, and Esther Witteveen (Amsterdam University Medical Center, location AMC, Amsterdam, The Netherlands); Marc J. Bonten, Olaf L. Cremer, Jos F. Frencken, Kirsten van de Groep, Peter M. Klein Klouwenberg, Maria E. Koster–Brouwer, David S. Ong, Meri R. Varkila and Diana M. Verboom (University Medical Center Utrecht, Utrecht, The Netherlands).

**Institutions:** University Medical Center Utrecht, Utrecht, The Netherlands; Amsterdam University Medical Centers, location Academic Medical Centre, University of Amsterdam, Amsterdam, The Netherlands.
