# Supplementary material for: Patients with hypothermic sepsis have a unique gene expression profile compared to patients with fever and sepsis
Source: J Cell Mol Med. 2022 Mar 1;26(7):1896–904. doi: 10.1111/jcmm.17156 (PMC8980902; doi:10.1111/jcmm.17156)
Supplement: Supplementary file 1 — Supplementary Material [file JCMM-26-1896-s001.doc]

SUPPLEMENTAL MATERIAL

**Patients with hypothermic sepsis have a unique gene expression profile compared to patients with fever and sepsis**

Matthew B.A. Harmon, MD, Brendon Scicluna, PhD, Maryse Wiewel, MD, PhD, Marcus J. Schultz, MD, PhD, Janneke Horn, MD, PhD, Olaf L. Cremer, MD, PhD, Tom van der Poll, MD, PhD, W. Joost Wiersinga, MD, PhD, Nicole P. Juffermans, MD, PhD, on behalf of the MARS consortium.

| **Supplemental table 1. Baseline characteristics and outcome of sepsis patients according to different temperatures** | | | | | |
| --- | --- | --- | --- | --- | --- |
|  | Hypothermia  N=67 | Normothermia  N= 75 | Fever  N=101 | Both  N=18 | P-value |
| **Demographics** |  |  |  |  |  |
| Age, years, mean [SD] | 68.1 [10.9] | 64.7 [14.6] | 60.0 [16.6] | 58.1 [17.3] | 0.002 |
| Gender, male (%) | 37 (55) | 34 (45) | 66 (65) | 12 (67) | 0.051 |
| BMI, kg/m2, mean [SD] | 25.5 [5.5] | 25.9 [6.4] | 27.0 [7.3] | 27.1 [7.2] | 0.498 |
| **Comorbidities** |  |  |  |  |  |
| Charlson score, median [IQR] | 5 [3-6] | 4 [3-7] | 4 [2-6] | 3 [2-4] | 0.003 |
| Chronic cardiovascular insufficiency (%) | 4 (6) | 2 (3) | 2 (2) | 4 (22) | 0.003 |
| Chronic renal insufficiency (%) | 11 (16) | 12 (16) | 6 (6) | 2 (11) | 0.109 |
| Congestive heart failure (%) | 3 (4) | 3 (4) | 4 (4) | 1 (6) | 1 |
| COPD (%) | 9 (13) | 16 (21) | 17 (17) | 2 (11) | 0.57 |
| Diabetes mellitus (%) | 19 (28) | 16 (21) | 16 (16) | 4 (22) | 0.276 |
| **Site of infection** |  |  |  |  |  |
| Pulmonary (%) | 28 (42) | 31 (41) | 50 (50) | 6 (33) | 0.524 |
| Abdominal (%) | 13 (19) | 19 (25) | 21 (21) | 3 (17) | - |
| Urinary tract (%) | 10 (15) | 10 (13) | 9 (9) | 3 (17) | - |
| Other (%) | 3 (4) | 9 (12) | 8 (8) | 3 (17) | - |
| Co-infection (%) | 13 (19) | 6 (8) | 13 (13) | 3 (17) | - |
| **Severity of disease first 24h** |  |  |  |  |  |
| Min temp first 24 h, mean [SD] | 35.0 [0.9] | 36.6 [0.5] | 37.2 [0.7] | 34.9 [0.7] | <0.0001 |
| Max temp first 24 h, mean [SD] | 37.1 [0.9] | 37.6 [0.9] | 39.3 [0.9] | 38.8 [0.9] | <0.0001 |
| APACHE IV score, median [IQR] ^a^ | 82 [71.5-104.5] | 80 [66.5-93.5] | 68 [55-84] | 92 [65.8-111.3] | <0.0001 |
| SOFA score, median [IQR] ^b^ | 9 [6-11] | 8 [4-10] | 7 [4-8] | 6 [4-11] | 0.001 |
| Acute kidney injury (%) | 38 (57) | 28 (37) | 31 (31) | 9 (50) | 0.009 |
| Renal replacement therapy (%) | 14 (21) | 4 (5) | 8 (8) | 4 (22) | 0.008 |
| Acute lung injury (%) | 21 (31) | 26 (35) | 30 (30) | 49 (22) | 0.751 |
| Shock (%) | 32 (48) | 26 (35) | 25 (25) | 7 (39) | 0.023 |
| **Clinical laboratory parameters first 24h** |  |  |  |  |  |
| WBC count max. ( x10^9/l), median [IQR] | 17.4 [10.6-27.8] | 15.5 [9.5-21.4] | 14.0 [9.9-17.9] | 15.0 [10.8-23.5] | 0.33 |
| Platelets min. (x10^9/l), median [IQR] | 186 [114-254] | 207 [129-297] | 208 [131-305] | 217 [146-248] | 0.458 |
| Lactate max. (mmol/l), median [IQR] | 3.2 [2.1-9.1] | 2.9 [1.7-5.2] | 2.5 [1.6-3.7] | 4.7 [1.95-6.38] | 0.036 |
| Prothrombin time max. (s), median [IQR] | 16.3 [14.0-22-2] | 14.8 [12.5-19.6] | 14.1 [12.1-16.8] | 15.8 [13.5-19.3] | 0.004 |
| Creatinine max. (μmol/l), median [IQR] | 114 [76-200] | 90 [65-162.5] | 99 [71.5-161.5] | 133 [82.3-234.5] | 0.114 |
| **Outcome** |  |  |  |  |  |
| ICU-mortality (%) | 21 (31) | 13 (17) | 9 (9) | 4 (22) | 0.003 |
| 30-day mortality (%) | 29 (43) | 20 (27) | 16 (16) | 4 (22) | 0.001 |
| 90-day mortality (%) | 35 (52) | 28 (37) | 22 (22) | 7 (39) | 0.002 |
| APACHE, acute physiology and chronic health evaluation; COPD, chronic obstructive pulmonary disease; IQR, interquartile range; SD, standard deviation; SOFA, sequential organ failure assessment; WBC, white blood cell. a Temperature not included in score  b Central nervous system not included in score due to large number of sedated patients | | | | | |

| **Supplemental table 2. Baseline characteristics in matched analysis for APACHE IV and SOFA score** | | | |
| --- | --- | --- | --- |
|  | Hypothermic  N=55 | Fever  N=55 | P-value |
| **Demographics** |  |  |  |
| Age, years, mean [SD] | 67.3 [11.6] | 61.8 [16.3] | 0.044 |
| Gender, male (%) | 30 (55) | 35 (64) | 0.455 |
| BMI, kg/m2, mean [SD] | 25.1 [5.6] | 27.3 [8.1] | 0.11 |
| **Comorbidities** |  |  |  |
| Charlson score, median [IQR] | 4 [3-6] | 4 [2-6] | 0.208 |
| Chronic cardiovascular insufficiency (%) | 4 (7) | 0 (0) | 0.139 |
| Chronic renal insufficiency (%) | 7 (13) | 5 (9) | 0.783 |
| Congestive heart failure (%) | 2 (4) | 1 (2) | 1 |
| COPD (%) | 6 (11) | 10 (18) | 0.42 |
| Diabetes mellitus (%) | 13 (24) | 9 (16) | 0.44 |
| **Site of infection** |  |  |  |
| Pulmonary (%) | 23 (42) | 27 (49) | 0.431 |
| Abdominal (%) | 10 (18) | 11 (20) | - |
| Urinary tract (%) | 9 (16) | 3 (5) | - |
| Other (%) | 2 (4) | 4 (7) | - |
| Co-infection (%) | 11 (29) | 10 (18) | - |
| **Severity of disease first 24h** |  |  |  |
| Min temp first 24 h, mean [SD] | 35.0 [0.9] | 37.2 [0.6] | <0.0001 |
| Max temp first 24 h, mean [SD] | 37.1 [0.9] | 39.2 [0.8] | <0.0001 |
| APACHE IV score, median [IQR] ^a^ | 81 [67.5-92.5] | 77 [64.5-90.5] | 0.76 |
| SOFA score, median [IQR] ^b^ | 8 [6-10] | 7 [5-9] | 0.258 |
| Acute kidney injury (%) | 28 (51) | 21 (38) | 0.259 |
| Renal replacement therapy (%) | 9 (16) | 7 (13) | 0.798 |
| Acute lung injury (%) | 15 (27) | 18 (32) | 0.682 |
| Shock (%) | 22 (40) | 21 (38) | 1 |
| **Clinical laboratory parameters first 24h** |  |  |  |
| WBC count max. ( x10^9/l), median [IQR] | 16.2 [10.4-27.8] | 14.2 [9.7-17.7] | 0.187 |
| Platelets min. (x10^9/l), median [IQR] | 188 [108-254] | 200 [121-293] | 0.365 |
| Lactate max. (mmol/l), median [IQR] | 2.9 [1.8-8.2] | 2.8 [2-5.2] | 0.4 |
| Prothrombin time max. (s), median [IQR] | 16.5 [15.0-22.5] | 14.6 [12.1-17.8] | 0.004 |
| Creatinine max. (μmol/l), median [IQR] | 112 [76-176] | 129 [77-174] | 0.805 |
| **Outcome** |  |  |  |
| ICU-mortality (%) | 14(25) | 6 (11) | 0.077 |
| 30-day mortality (%) | 21 (38) | 10 (18) | 0.041 |
| 90-day mortality (%) | 26 (47) | 12 (22) | 0.008 |
| APACHE, acute physiology and chronic health evaluation; COPD, chronic obstructive pulmonary disease; IQR, interquartile range; SD, standard deviation; SOFA, sequential organ failure assessment; WBC, white blood cell. a Temperature not included in score  b Central nervous system not included in score due to large number of sedated patients | | | |

**Supplementary Table 3.** List of differentially expressed protein-coding genes in the matched cohort.

| **Gene symbol** | **Affymetrix U219 probe ID** | **Log2 Fold Change** | **T statistic** | **P Value** | **Adjusted P Value** |
| --- | --- | --- | --- | --- | --- |
| CIRBP | 11715693_s_at | 0.67 | 6.46 | 1.17E-09 | 2.88E-05 |
| ALDH2 | 11715627_x_at | 0.79 | 5.82 | 3.00E-08 | 0.00037 |
| TEP1 | 11723894_a_at | -0.69 | -5.49 | 1.54E-07 | 0.00084 |
| ATG2A | 11723601_a_at | 0.55 | 5.47 | 1.70E-07 | 0.00084 |
| TBCK | 11720266_a_at | -0.46 | -5.36 | 2.81E-07 | 0.00102 |
| TMED4 | 11759738_at | -0.39 | -5.35 | 2.90E-07 | 0.00102 |
| C1orf63 | 11721441_x_at | -0.69 | -5.30 | 3.68E-07 | 0.00106 |
| ORM2 | 11746256_x_at | 0.90 | 5.27 | 4.32E-07 | 0.00106 |
| CTDSP2 | 11716957_x_at | 0.51 | 5.22 | 5.32E-07 | 0.00118 |
| PTPLAD2 | 11745234_a_at | -0.45 | -5.18 | 6.46E-07 | 0.00118 |
| TTC9C | 11721190_a_at | -0.47 | -5.17 | 6.73E-07 | 0.00118 |
| PLIN5 | 11737160_at | 0.58 | 5.04 | 1.24E-06 | 0.00181 |
| NDUFB3 | 11763960_a_at | -0.59 | -5.04 | 1.25E-06 | 0.00181 |
| RBBP5 | 11760373_at | -0.28 | -5.01 | 1.39E-06 | 0.00191 |
| RBM3 | 11744146_x_at | 0.82 | 4.97 | 1.70E-06 | 0.00207 |
| THAP2 | 11730969_at | -0.23 | -4.96 | 1.76E-06 | 0.00207 |
| ZNF271 | 11750557_x_at | -0.35 | -4.90 | 2.26E-06 | 0.00254 |
| LRRC8C | 11723592_at | -0.51 | -4.88 | 2.56E-06 | 0.00264 |
| DCLRE1C | 11762018_at | -0.47 | -4.86 | 2.72E-06 | 0.00264 |
| HTATIP2 | 11718979_a_at | -0.39 | -4.86 | 2.79E-06 | 0.00264 |
| PER1 | 11717168_a_at | 0.54 | 4.84 | 2.99E-06 | 0.00273 |
| SOD1 | 11757619_x_at | -0.52 | -4.79 | 3.75E-06 | 0.00301 |
| FGD4 | 11726395_s_at | 0.33 | 4.79 | 3.79E-06 | 0.00301 |
| ATXN3 | 11726361_a_at | -0.33 | -4.78 | 3.96E-06 | 0.00305 |
| C12orf5 | 11719731_at | -0.43 | -4.74 | 4.68E-06 | 0.00349 |
| VSIG4 | 11747010_a_at | 0.91 | 4.67 | 6.26E-06 | 0.00430 |
| PTPRJ | 11728972_a_at | 0.49 | 4.67 | 6.28E-06 | 0.00430 |
| CTSC | 11731465_a_at | -0.41 | -4.61 | 8.01E-06 | 0.00493 |
| RER1 | 11744509_a_at | -0.24 | -4.56 | 1.01E-05 | 0.00591 |
| HSPH1 | 11753964_x_at | -0.80 | -4.55 | 1.04E-05 | 0.00591 |
| JTB | 11744494_x_at | -0.21 | -4.55 | 1.05E-05 | 0.00591 |
| WIPF1 | 11759628_at | 0.37 | 4.49 | 1.37E-05 | 0.00728 |
| CACYBP | 11727786_x_at | -0.45 | -4.48 | 1.39E-05 | 0.00728 |
| CNIH4 | 11758808_a_at | -0.57 | -4.48 | 1.42E-05 | 0.00728 |
| CLEC2D | 11730321_a_at | -0.33 | -4.46 | 1.49E-05 | 0.00742 |
| MRPL33 | 11757411_x_at | -0.38 | -4.46 | 1.51E-05 | 0.00742 |
| HSP90AB1 | 200064_PM_at | -0.55 | -4.45 | 1.59E-05 | 0.00768 |
| ORM1 | 11758794_x_at | 1.10 | 4.43 | 1.74E-05 | 0.00815 |
| FKBP4 | 11736021_a_at | -0.44 | -4.43 | 1.75E-05 | 0.00815 |
| NUP214 | 11758849_at | -0.41 | -4.40 | 1.93E-05 | 0.00851 |
| RIPK1 | 11756807_a_at | 0.34 | 4.36 | 2.32E-05 | 0.00985 |
| IFNAR1 | 11723425_at | -0.44 | -4.35 | 2.37E-05 | 0.00992 |
| BCKDK | 11743293_a_at | 0.32 | 4.34 | 2.52E-05 | 0.01002 |
| KIAA1841 | 11732797_x_at | -0.29 | -4.32 | 2.76E-05 | 0.01078 |
| CCT5 | 11747068_x_at | -0.32 | -4.28 | 3.18E-05 | 0.01190 |
| KLF9 | 11717327_at | 0.64 | 4.28 | 3.19E-05 | 0.01190 |
| MRPS5 | 11763290_at | -0.32 | -4.25 | 3.57E-05 | 0.01293 |
| UTP14C | 11722485_a_at | -0.37 | -4.24 | 3.79E-05 | 0.01354 |
| AP3M1 | 11729628_a_at | -0.33 | -4.23 | 3.91E-05 | 0.01378 |
| USP15 | 11759627_at | 0.53 | 4.22 | 4.06E-05 | 0.01390 |
| AMPH | 11724473_a_at | 0.56 | 4.21 | 4.28E-05 | 0.01404 |
| STYXL1 | 11723993_a_at | -0.42 | -4.21 | 4.28E-05 | 0.01404 |
| ACTA2 | 11722352_s_at | -0.58 | -4.21 | 4.30E-05 | 0.01404 |
| PIGF | 11737862_at | -0.22 | -4.20 | 4.37E-05 | 0.01404 |
| TRAF3IP3 | 11763426_a_at | -0.38 | -4.17 | 4.94E-05 | 0.01542 |
| OMA1 | 11731585_a_at | -0.50 | -4.12 | 6.03E-05 | 0.01834 |
| AHSA1 | 11716083_a_at | -0.35 | -4.12 | 6.13E-05 | 0.01843 |
| ASB6 | 11726924_a_at | -0.24 | -4.11 | 6.35E-05 | 0.01885 |
| SYAP1 | 11744428_x_at | 0.28 | 4.10 | 6.42E-05 | 0.01885 |
| PNPLA1 | 11735905_x_at | -0.30 | -4.10 | 6.55E-05 | 0.01888 |
| ITPR1 | 11739564_s_at | -0.43 | -4.10 | 6.61E-05 | 0.01888 |
| KLHL6 | 11727572_s_at | -0.55 | -4.09 | 6.82E-05 | 0.01888 |
| PAPOLA | 11719009_a_at | -0.30 | -4.09 | 6.83E-05 | 0.01888 |
| SP100 | 11737256_at | -0.40 | -4.09 | 6.89E-05 | 0.01888 |
| MIER1 | 11729389_a_at | -0.28 | -4.08 | 7.01E-05 | 0.01899 |
| DPH3 | 11733040_a_at | -0.40 | -4.06 | 7.67E-05 | 0.01956 |
| MTRF1L | 11739182_s_at | -0.37 | -4.06 | 7.69E-05 | 0.01956 |
| TMEM79 | 11727517_a_at | -0.24 | -4.01 | 9.20E-05 | 0.02197 |
| GRB10 | 11741924_a_at | 0.55 | 4.01 | 9.39E-05 | 0.02197 |
| KIAA0907 | 11719331_s_at | -0.37 | -4.00 | 9.43E-05 | 0.02197 |
| PTGES3 | 11743372_s_at | -0.37 | -4.00 | 9.45E-05 | 0.02197 |
| ACBD6 | 11757520_s_at | 0.21 | 4.00 | 9.68E-05 | 0.02207 |
| C11orf82 | 11725161_a_at | -0.55 | -3.99 | 9.83E-05 | 0.02207 |
| SZRD1 | 11716537_s_at | -0.36 | -3.97 | 0.00011 | 0.02318 |
| SSU72 | 11763451_s_at | 0.32 | 3.96 | 0.00011 | 0.02393 |
| DMXL1 | 11724711_a_at | 0.22 | 3.95 | 0.00011 | 0.02423 |
| SNRNP27 | 11743660_x_at | -0.42 | -3.95 | 0.00012 | 0.02423 |
| MAOA | 11745923_x_at | 0.52 | 3.95 | 0.00012 | 0.02423 |
| RNF20 | 11758375_s_at | -0.34 | -3.94 | 0.00012 | 0.02500 |
| CITED4 | 11724948_at | 0.23 | 3.93 | 0.00013 | 0.02524 |
| TRMT1L | 11728212_at | -0.37 | -3.92 | 0.00013 | 0.02560 |
| TMX2 | 11715612_s_at | -0.35 | -3.92 | 0.00013 | 0.02570 |
| PDZD11 | 11716585_a_at | -0.23 | -3.92 | 0.00013 | 0.02570 |
| NOL12 | 11725339_at | -0.24 | -3.89 | 0.00014 | 0.02738 |
| HSPA6 | 11723050_a_at | -0.50 | -3.89 | 0.00015 | 0.02752 |
| HIST2H2BE | 11717090_a_at | -0.54 | -3.88 | 0.00015 | 0.02791 |
| AIP | 11715980_at | -0.32 | -3.88 | 0.00015 | 0.02791 |
| MKNK2 | 11739147_a_at | -0.50 | -3.88 | 0.00015 | 0.02791 |
| THBS1 | 11758842_at | 0.50 | 3.88 | 0.00015 | 0.02791 |
| CCT3 | 11716382_a_at | -0.36 | -3.88 | 0.00015 | 0.02791 |
| ACSL5 | 11723946_a_at | -0.43 | -3.87 | 0.00016 | 0.02845 |
| SLC6A6 | 11721991_a_at | 0.32 | 3.86 | 0.00016 | 0.02892 |
| FANCD2 | 11736547_a_at | 0.39 | 3.85 | 0.00017 | 0.02950 |
| ZNF784 | 11725144_a_at | 0.25 | 3.85 | 0.00017 | 0.02994 |
| ERGIC1 | 11760575_at | 0.22 | 3.84 | 0.00018 | 0.03017 |
| ZBTB16 | 11729371_a_at | 0.81 | 3.84 | 0.00018 | 0.03017 |
| KBTBD11 | 11721566_s_at | 0.46 | 3.83 | 0.00018 | 0.03069 |
| MED31 | 11734794_a_at | -0.36 | -3.83 | 0.00018 | 0.03069 |
| FAM96B | 11715907_s_at | -0.29 | -3.81 | 0.00019 | 0.03224 |
| ECHDC3 | 11728810_a_at | 0.71 | 3.81 | 0.00020 | 0.03233 |
| DTD1 | 11746103_a_at | 0.19 | 3.80 | 0.00020 | 0.03274 |
| LRP3 | 11724852_s_at | 0.35 | 3.79 | 0.00021 | 0.03362 |
| C9orf72 | 11737176_at | 0.30 | 3.79 | 0.00021 | 0.03363 |
| TEFM | 11731119_a_at | -0.43 | -3.79 | 0.00021 | 0.03363 |
| RNF145 | 11745856_x_at | 0.41 | 3.79 | 0.00021 | 0.03378 |
| NEU1 | 11715838_a_at | -0.33 | -3.78 | 0.00022 | 0.03396 |
| C2orf42 | 11723835_at | -0.28 | -3.78 | 0.00022 | 0.03485 |
| SDC4 | 11717638_at | 0.22 | 3.77 | 0.00023 | 0.03485 |
| TRAPPC2 | 11724432_x_at | -0.29 | -3.77 | 0.00023 | 0.03485 |
| CCDC88C | 11760013_a_at | -0.31 | -3.77 | 0.00023 | 0.03485 |
| YIPF5 | 11733165_s_at | -0.49 | -3.77 | 0.00023 | 0.03485 |
| SAP30 | 11727476_s_at | 0.36 | 3.77 | 0.00023 | 0.03485 |
| DDX5 | 11733798_at | -0.29 | -3.76 | 0.00024 | 0.03513 |
| SUV420H1 | 11721416_a_at | 0.26 | 3.75 | 0.00024 | 0.03571 |
| SNRNP48 | 11758437_s_at | -0.28 | -3.75 | 0.00024 | 0.03571 |
| TRMT13 | 11761560_x_at | -0.44 | -3.75 | 0.00024 | 0.03571 |
| TBC1D20 | 11728740_a_at | -0.27 | -3.75 | 0.00025 | 0.03574 |
| SLC24A6 | 11750663_x_at | 0.18 | 3.75 | 0.00025 | 0.03582 |
| HSPA4 | 11758770_at | -0.31 | -3.74 | 0.00025 | 0.03617 |
| GRK6 | 11733846_a_at | 0.34 | 3.74 | 0.00026 | 0.03635 |
| DAAM2 | 11724178_a_at | 0.93 | 3.73 | 0.00026 | 0.03672 |
| ZNF512 | 11717882_a_at | -0.27 | -3.72 | 0.00027 | 0.03742 |
| CXorf65 | 11729520_at | 0.36 | 3.72 | 0.00027 | 0.03742 |
| CYB561D1 | 11722960_a_at | -0.23 | -3.71 | 0.00028 | 0.03827 |
| C1orf21 | 11755335_a_at | -0.33 | -3.71 | 0.00029 | 0.03884 |
| RAB10 | 11742995_at | -0.23 | -3.70 | 0.00029 | 0.03899 |
| CEPT1 | 11754503_a_at | -0.43 | -3.70 | 0.00030 | 0.03971 |
| TLR10 | 11746407_x_at | -0.46 | -3.70 | 0.00030 | 0.03971 |
| CX3CR1 | 11723048_at | -0.80 | -3.68 | 0.00031 | 0.04100 |
| TXNL4B | 11722382_a_at | -0.27 | -3.68 | 0.00031 | 0.04100 |
| ZFC3H1 | 11743317_at | -0.32 | -3.68 | 0.00032 | 0.04121 |
| CHMP4B | 11716497_at | 0.20 | 3.67 | 0.00033 | 0.04190 |
| FUZ | 11747568_a_at | 0.18 | 3.66 | 0.00034 | 0.04311 |
| SLC36A4 | 11743091_x_at | 0.51 | 3.65 | 0.00035 | 0.04392 |
| PNPLA2 | 11730221_at | 0.18 | 3.64 | 0.00036 | 0.04527 |
| P2RX7 | 11736064_x_at | -0.24 | -3.64 | 0.00037 | 0.04661 |
| GNS | 11716752_x_at | -0.35 | -3.63 | 0.00039 | 0.04775 |
| CRNKL1 | 11730751_s_at | -0.39 | -3.62 | 0.00040 | 0.04873 |
| ST20 | 11744313_a_at | -0.39 | -3.61 | 0.00040 | 0.04892 |
| C20orf111 | 11759490_at | -0.31 | -3.61 | 0.00041 | 0.04904 |
| KHNYN | 11721163_a_at | 0.32 | 3.61 | 0.00041 | 0.04934 |

Supplemental figure 1


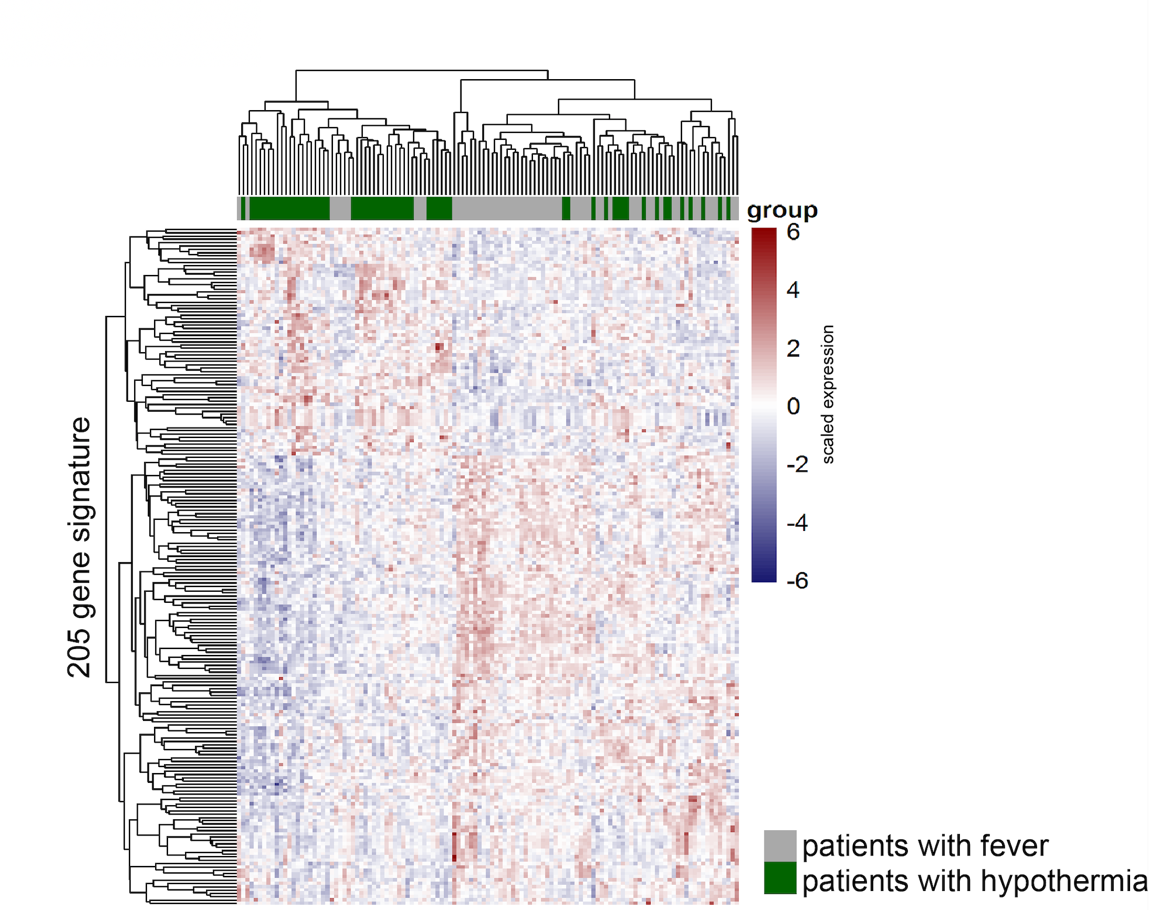


**Figure legend**

Figure 1. Unsupervised heatmap representation of the 205 significantly altered genes from whole blood leukocyte microarray analysis in hypothermic septic patients versus febrile septic patients.

Supplemental figure 2

**Figure legend**

Figure 2. (A) Volcano plot of gene expression profiles of hypothermic septic patients compared to febrile patients in the unmatched cohort. The volcano plot shows the differences in gene expression in hypothermia compared to fever (x-axis) and multiple-comparison adjusted p values for hypothermia compared to fever (y-axis). (B) shows the up- and downregulated canonical pathways associated with the gene expression profiles in hypothermia compared to fever.
